# Draft genome sequence of endometrial *Schaalia turicensis* strain R31 from a patient with adenomyosis

**DOI:** 10.1128/mra.00961-25

**Published:** 2025-11-24

**Authors:** Nicole Jimenez, Matthew Hawkins, Bonnie Hurwitz, Melissa M. Herbst-Kralovetz

**Affiliations:** 1 Department of Obstetrics and Gynecology, College of Medicine-Phoenix, University of Arizona 42283, Phoenix, Arizona, USA; 2 Department of Basic Medical Sciences, College of Medicine-Phoenix, University of Arizona 42283 , Phoenix, Arizona, USA; 3 Department of Biosystems Engineering, University of Arizona 8041, Tucson, Arizona, USA; 4 University of Arizona Cancer Center 613590, Tucson, Arizona, USA; Loyola University Chicago, Chicago, Illinois, USA

**Keywords:** Actinomyces, *Schaalia turicensis*, female reproductive tract, uterus, endometrium, adenomyosis

## Abstract

The endometrial *Schaalia turicensis* strain R31 genome was isolated from a post-hysterectomy endometrial glycerol swab obtained from a woman diagnosed with adenomyosis. Here, we report a 1.97 Mb complete draft genome sequence of this strain.

## ANNOUNCEMENT

*Actinomyces turicensis* was reclassified (1) as *Schaalia turicensis*. These Gram-positive facultative anaerobes exist in the human oral (2, 3), gut (4), and urogenital tracts (5–7). *S. turicensis* is an opportunistic pathogen with cases involving urethritis (5), bacteremia (8), meningitis (9), soft tissue infections (10), and wound infections (11).

In this announcement, we describe the draft genome of *S. turicensis* R31, which was isolated from the endometrium, thereby expanding our understanding of *S. turicensis* in gynecologic health.

This isolate was obtained from an endometrial swab of a patient diagnosed with adenomyosis, in which endometrial-like tissue grows into the myometrium. This was conducted as part of a multi-omic study investigating patients undergoing hysterectomy (12–14). The Institutional Review Board of the University of Arizona approved this study (reference no. 1708726047), and written informed consent was obtained from the study participant. Swabs were frozen in Amies transport media with 10% glycerol. Serial dilutions were performed to isolate the bacteria under anaerobic conditions at 37°C on Tryptic Soy Agar supplemented with 5% sheep’s blood for 48 h. Bacterial DNA was extracted using the Qiagen DNeasy PowerSoil Pro Kit (MO BIO Laboratories, Carlsbad) and sequenced at the University of Arizona PANDA Core for Genomics and Microbiome Research. Paired-end sequencing was performed using Illumina’s PCR-Free Library Prep Kit and the NextSeq 1000 Platform (300-cycle) with read length of 35–151 bp. Trimmomatic (v0.39, ILLUMINACLIP:TruSeq3-PE-2.fa:2:30:10, SLIDINGWINDOW:4:20, MINLEN:100, HEADCROP:15) (15) improved read quality and was assessed using FastQC (v0.11.9) (16). Kraken2 (v2.1.3) (17) and Bracken (v2.8) (17) were used for species-level read classification based on the k2_pluspf database (downloaded on 2023-06-05) (18). Krakentools (v1.2) (17) (extract_kraken_reads.py, --taxid 9606, --include-children) was used to separate human from microbial reads. Assembly was performed using Unicycler (v16.0) (19), followed by quality checks with Checkm2 (v1.0.1, -m 500) (20) and Quast (v5.2.0) (21). Annotation was performed using PGAP (v 6.1) (22). Default parameters were used for all tools unless otherwise specified. All code is available on GitHub (https://github.com/hurwitzlab/vaginal_genome_assembly). Genomic analyses were performed on the Bacterial and Viral Bioinformatics Resources Center website (23).

The draft genome of *S. turicensis* R31 comprises 38 contigs, totaling 1,973,689 base pairs, with a GC content of 56.83%. The assembly exhibits moderate contiguity with an N50 of 102,621 bp. Annotation revealed 1,750 coding sequences, 47 tRNA genes, and three rRNA genes. Of these, 229 were annotated as hypothetical proteins, while 1,521 had functional assignments.

Subsystem classification identified metabolism (196 genes) as the largest functional group (Table 1). Amino acid metabolism made up 34.7% of the genome’s capability. Protein comparisons with public *S. turicensis* strains indicated 438 shared protein families and 24 protein families unique to R31 (Table 1). Two of these were related to cell wall/invasion-associated proteins and histone acetyltransferase, while 22 families were of hypothetical origin (Table 1). Antibiotic resistance genes (24 genes) were identified, which may be crucial for treating actinomycosis (Table 1).

**TABLE 1:** Genome characteristics table for *S. turicensis* R31^*a*^

Genome name	*S. turicensis* R31
Isolate information	
Isolation source	Endometrium
Health status	Adenomyosis
Strain identity	
KRAKEN2 taxonomy	*Schaalia turicensis*
Average nucleotide identity to representative strain (%)	95.95
Average nucleotide identity to Clade 1 (mean) (%)	96.76
Average nucleotide identity to Clade 2 (mean) (%)	67.97
Genome characteristics	
Raw reads	4058498
Genome size (bp)	1,973,689
Number of contigs	38
Contig N50 (bp)	102,621
Contig L50	6
GC (%)	56.83%
Genome coverage	200×
Number of 5S rRNA	1
Number of 16S rRNA	1
Number of 23S rRNA	1
Number of tRNAs	47
Number of CDS	1,750
Number of CDS with functional assignments	1,521
Number of unique protein families	24
Unique protein family IDs (PGFAMs) – hypothetical	PGF_12915410, PGF_06376995, PGF_12422020, PGF_11667696, PGF_00274664, PGF_01749377, PGF_00426553, PGF_00273925, PGF_12868907, PGF_08271889, PGF_02376408, PGF_00978629, PGF_06147024, PGF_02629833, PGF_04698765, PGF_00116012, PGF_05143424, PGF_00208348, PGF_05574635, PGF_00263229, PGF_02376410
Unique protein family IDs (PGFAMs) – non-hypothetical	PGF_00041234, PGF_10350959, PGF_10402854
Number of antibiotic resistance genes (PATRIC)	24
Antibiotic resistance gene IDs	Alr, Ddl, Dfr, dxr, EF-G, EF-Tu, folA, folP, GdpD, gidB, gyrA, gyrB, iso-tRNA, LpqB, MtrA, MtrB, MurA, PgsA, rpoB, rpoC, rho, S10p, S12p

^*a*^ The table includes isolate information, including the source and health status of the patient from which the isolate was obtained. Further strain identity information, including taxonomy classification based on two databases (Kraken2) and average nucleotide identity compared to all *S. turicensis *genomes, genomes within Clade 1, and genomes within Clade 2, is highlighted in the phylogenetic tree. In addition, genomic characteristics were obtained from annotated and evaluated with BV-BRC, including genome size, number of contigs, contig N50, contig L50, GC content, genome coverage, number of 5S rRNA, 16S rRNA, 23S rRNA, tRNAs, CDS, and CDS with functional assignments. The data also include the number of unique protein families specific to the R31 genome and their corresponding PATRIC cross-genus family IDs, as well as the number of antibiotic resistance genes identified by PATRIC, along with their gene IDs. Methods and version numbers for genome assembly can be found at https://github.com/hurwitzlab/vaginal_genome_assembly.

**Fig 1:**
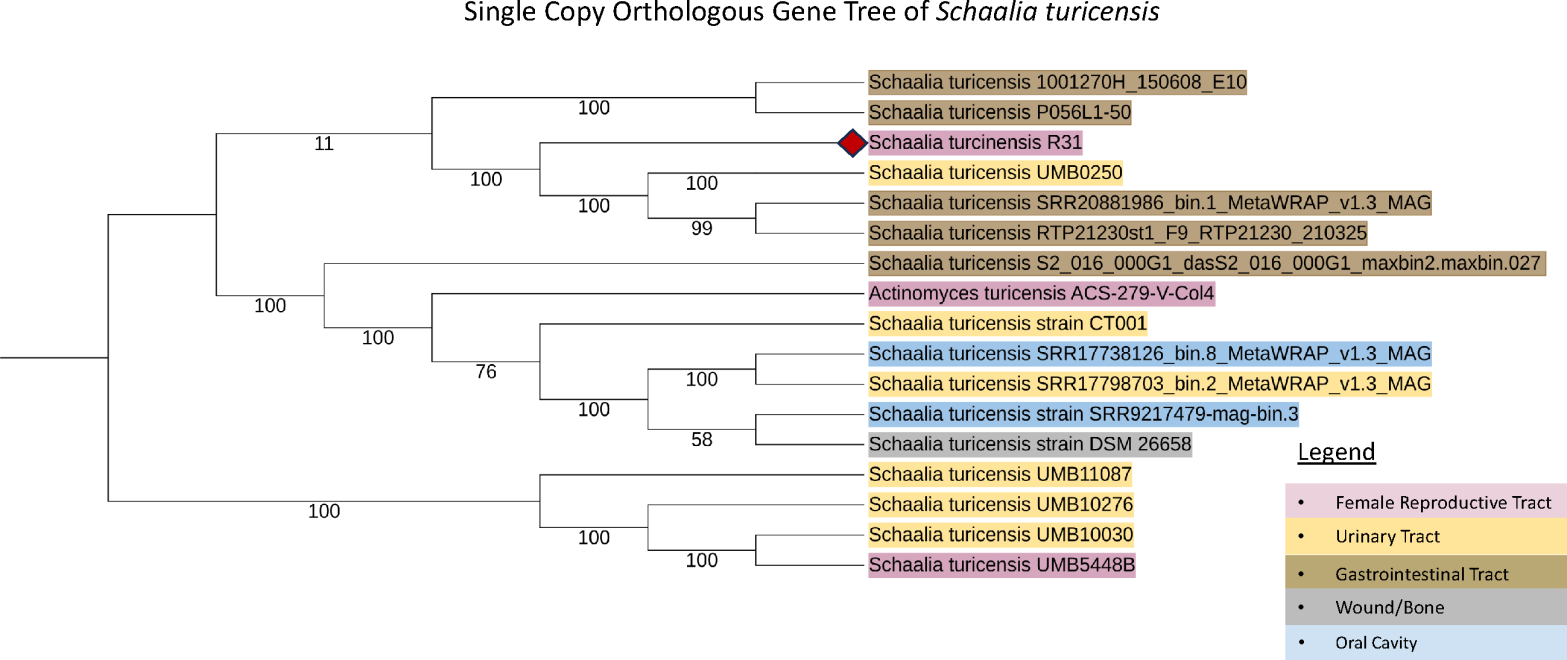
Single-copy orthologous phylogenetic tree among 16 publicly available *S. turicensis* strains and R31. Single-copy orthologous genes were used to create the bacterial genome tree (*n* = 278) and were aligned by codon using RaXML as part of the BV-BRC bacterial genome tree pipeline using default parameters. The tree comprises 11 whole-genome assemblies and five metagenome-assembled genomes of S. turicensis. *S. turicensis* R31 is the strain discussed in this microbial resource announcement and indicated by a red diamond. The colors of *S. turicensis* strain names are based on the environmental source from which the genomes originated.

Kraken2 classification confirms the R31 identity within the *Schaalia* genus. Additional strain-specific phylogenetic analysis revealed a non-specific placement based on isolation source (Fig. 1), with an average nucleotide identity of 96.8% to other *S. turicensis* in Clade 1 and 68% in Clade 2 (Table 1). Together, these findings highlight the fundamental genomic capabilities of the endometrial *S. turicensis* R31 genome, laying a foundation for investigating its role in gynecologic health.

## Data Availability

The draft genome sequence of Schaalia turicensis strain R31 has been deposited in the Sequence Read Archive (SRA) under accession number SRR35785846, after filtering out sequences below 200 bp. This genome is part of a larger BioProject (PRJNA1036657) associated with 16S rRNA level data of vaginal (SAMN38145775) and rectal (SAMN38145877) microbiomes. The genome BioSample identification is SAMN50643546. The genome annotation can also be found at GenBank GCA_052216585.1 and additional genome assembly, annotation, protein family, and phylogenetic analysis information at BV-BRC (https://www.bv-brc.org/workspace/jimeneznr@patricbrc.org/Schaalia). Additional assembly information and parameters utilized can be found at https://github.com/hurwitzlab/vaginal_genome_assembly.
